# Influence of postoperative ectopic inner foveal layer on visual function after removal of idiopathic epiretinal membrane

**DOI:** 10.1371/journal.pone.0259388

**Published:** 2021-11-04

**Authors:** Bo Hee Kim, Dong Ik Kim, Ki Woong Bae, Un Chul Park

**Affiliations:** Department of Ophthalmology, Seoul National University College of Medicine, Seoul, Korea; National Yang-Ming University Hospital, TAIWAN

## Abstract

**Purpose:**

To investigate the functional and anatomical parameters and their postoperative changes according to the ectopic inner foveal layer (EIFL) staging scheme for idiopathic epiretinal membrane (ERM).

**Methods:**

In this prospective study, patients with idiopathic ERM underwent pars plana vitrectomy and ERM removal, and were followed-up for 6 months. The associations of EIFL with pre- and postoperative functional and anatomical parameters were analyzed.

**Results:**

A total of 84 eyes (84 patients) were included: 39 (46.4%), 33 (39.3%), and 12 (14.3%) as EIFL stages 2, 3, and 4, respectively. At 6 months after surgery, the mean best-corrected visual acuity (BCVA) significantly improved in all EIFL stages (*P* ≤ 0.003); however, metamorphopsia improved only in eyes with EIFL stage 2 (*P* = 0.039) and 3 (*P* = 0.011). The aniseikonia and foveal avascular zone (FAZ) area showed no significant postoperative changes in any of the EIFL stages. Both preoperatively and during 6 months after surgery, the EIFL stage showed a significant correlation with BCVA (*P* ≤ 0.033), metamorphopsia (*P* ≤ 0.008), central macular thickness (*P* < 0.001), and FAZ parameters (*P* ≤ 0.016) at each time point, but not with aniseikonia. Significant correlations of EIFL thickness with BCVA (*P* = 0.028) and metamorphopsia (*P* = 0.006) before surgery were not persistent after surgery.

**Conclusion:**

Both pre- and postoperatively, the staging of EIFL, rather than its thickness, is a simple and adequate surrogate marker for visual acuity and metamorphopsia in eyes with idiopathic ERM.

## Introduction

Epiretinal membrane (ERM) is one of the most common retinal disorders with a higher prevalence in elderly people and generally idiopathic [1]. It is characterized by contractile avascular fibrocellular proliferation on the inner surface of the retina deforming its normal structure, and can result in a decrease in central vision, metamorphopsia, macropsia, or rarely micropsia [2,3].

With recent advances in optical coherence tomography (OCT) technology, ERM can be easily diagnosed and evaluated during follow-up. Based on spectral-domain OCT findings, several biomarkers focusing on outer retinal changes, such as the integrity of ellipsoid and interdigitation zone band or photoreceptor outer segment length [4–6], have been proposed to predict postoperative visual outcome after ERM removal. More recently, due to the limited efficacy of outer retinal biomarkers to predict visual prognosis [7], inner retinal biomarkers such as inner retinal layer thickness [8], inner retinal irregularity index [9], or presence of ectopic inner foveal layers (EIFL) [10] have been increasingly reported to have an impact on visual acuity in eyes with ERM. Govetto et al. suggested that the presence of continuous EIFL in ERM, as an essential element of a novel OCT-based grading scheme showing ERM progression, is associated with significant vision loss [10]. In addition, EIFL staging has surgical significance as a prognostic factor for visual acuity after ERM removal, and may be considered significantly during the decision process for ERM surgery [7,11].

However, the association of the EIFL staging scheme with functional parameters other than visual acuity, such as metamorphopsia and aniseikonia, has not been evaluated. In addition, the influence of EIFL on changes in the foveal avascular zone (FAZ) after ERM removal is unknown. In this study, we analyzed the pre- and post-operative functional and anatomical parameters according to the EIFL staging scheme in a prospective cohort of patients with idiopathic ERM who underwent pars plana vitrectomy (PPV) with ERM removal.

## Materials and methods

### Patients

This prospective study enrolled consecutive patients who underwent PPV for the treatment of idiopathic ERM at Seoul National University Hospital from January 2019 to April 2020. The study protocol was reviewed and approved by the Institutional Review Board of the Seoul National University Hospital (IRB no: 1901-159-1006). All study procedures adhered to the tenets of the Declaration of Helsinki, and written informed consent was obtained from all patients before surgery to participate in this study. The ERM was diagnosed clinically by slit-lamp ophthalmoscopy using a 90-diopter lens and spectral-domain OCT. The exclusion criteria were as follows: (1) patients with ERM secondary to other retinal diseases, such as uveitis, retinal detachment, and retinal vascular diseases; (2) any ocular disease that can affect central visual function; (3) history of previous vitreoretinal surgery; and (4) follow-up period of less than 6 months after PPV.

### Ophthalmic examination

Before the surgery, all patients underwent a complete ophthalmic examination, including slit-lamp biomicroscopy, fundus examination, best-corrected visual acuity (BCVA), intraocular pressure, spectral-domain OCT, and OCT angiography (OCTA). The BCVA was converted into the logarithm of the minimal angle of resolution (logMAR) values for statistical analysis. For macular function evaluation, tests for metamorphopsia and aniseikonia were also performed.

Spectral-domain OCT examination was performed using a Cirrus HD (Carl Zeiss Meditec, Dublin, CA, USA) for all quantitative and qualitative measurements. The severity of ERM was graded according to the Govetto et al’ EIFL classification scheme [10]; Stage 1, a mild ERM with no anatomical distortion and preserved foveal depression; Stage 2, an ERM with loss of the foveal depression, but well-defined all retinal layers; Stage 3, the presence of a continuous inner nuclear layer (hyporeflective) and inner plexiform layer (hyperreflective) band covering the fovea; and Stage 4, the presence of EIFL and disrupted all retinal layers. The thickness of the EIFL, which was defined as the distance between the inner border of the outer nuclear layer and the internal limiting membrane (ILM) at the foveal center, was measured manually using a caliper provided by the machine, and measurements from the horizontal and vertical scans were averaged for analysis. Central macular thickness (CMT) was obtained from the central 1-mm subfield in the macular thickness map.

A 6 × 6 mm OCTA image centered on the fovea was obtained using a swept-source OCTA (Plex Elite 9000; Carl Zeiss Meditec, Dublin, CA). Images with a signal intensity of ≥ 7 /10 were defined as suitable images. Using the Advanced Retina Imaging Zeiss Macular Density Algorithm v 0.7.1, which is a prototype, proprietary algorithm available in Carl Zeiss online analysis that extracts the FAZ boundary and allows quantification of FAZ area at the superficial capillary plexus in the macular area, superficial FAZ area, superficial FAZ perimeter, and superficial FAZ circularity were obtained.

Metamorphopsia was evaluated using M-CHARTS (Inami Co., Tokyo, Japan) as previously reported [12]. Briefly, a straight line was presented to a patient, and if the line was reported as straight, the M-score was recorded as 0. If not, a dotted line with intervals ranging from 0.2° to 2.0° visual angle was shown beginning with a line comprising a small separation of dots until the patient reported that the dotted line appeared straight. The minimum angle of the dots that appeared straight was determined as the patient’s M-score. This test was performed using both horizontal and vertical lines, and the results were averaged for the analysis. Aniseikonia was evaluated using the New Aniseikonia Test (NAT version 3; Handaya, Tokyo, Japan) as previously reported [13]. Briefly, patients viewed pairs of adjacent calibrated hemi-circle targets with one red and one green color using a pair of glasses with a red filter and green filter in each eye. Targets were presented in 1% increments in the size difference between the two hemi-circle targets from 0% to 24%. The percentage at which the patient perceived both semi-circles as being of the same size was recorded as the patient’s aniseikonia score. Measurements were performed with semi-circles oriented both horizontally and vertically to obtain horizontal and vertical aniseikonia scores, and were averaged for analysis. For both the M-score and aniseikonia scores, testing was repeated three times to confirm its reliability. Analysis of the aniseikonia score was conducted only in patients with unilateral ERM.

### Surgical procedures

Pars plana vitrectomy was indicated in patients with visual acuity reductions or visual disturbances, such as metamorphopsia. Patients with ERM of EIFL stage 1 were not considered for surgery in this study. All surgeries were performed by a single experienced surgeon (UCP). A standard small gauge (23 G or 25 G) three-port PPV was performed, and combined phacoemulsification and intraocular lens implantation were performed in eyes with significant cataracts. The presence of posterior vitreous detachment was assessed intraoperatively and induced if not present. Using microforceps, both ERM and ILM were removed to the vascular arcades in all cases. Indocyanine green dye was applied over the retinal surface to enhance the visualization of the ILM during peeling. All patients received a standard postoperative protocol of topical antibiotic and anti-inflammatory medications, and the results at 3 and 6 months after PPV, including BCVA, spectral-domain OCT, OCTA, metamorphopsia, and aniseikonia, were analyzed.

### Statistical analysis

Continuous variables such as BCVA (logMAR), CMT, EIFL thickness, and OCTA parameters were expressed as mean ± standard deviation; categorical variables such as sex and EIFL stage were expressed as frequency and percentage. Categorical variables were compared using the chi-square test. For quantitative comparative variables, Kruskal-Wallis test was performed to compare participants between each EIFL ERM stage. The relationships between visual functions and the EIFL stage were examined by the Spearman rank correlation test. Partial correlation analysis was conducted to evaluate the influence of possible confounding variables. Statistical significance was set at *P* value < 0.05. For the multiple post hoc comparison between the EIFL stages using Bonferroni, *P* < 0.0167 was considered significant. The analyses were performed using SPSS (version 23.0; IBM Corp., Armonk, NY).

## Results

A total of 84 eyes (84 patients) were included in this study, and their baseline characteristics are summarized in Table 1. Among all patients, 24 (28.6%) had bilateral ERMs and were excluded from the analysis for aniseikonia. The mean age at the time of surgery was 68.7 ± 7.9 years (range, 51–88), and 57 patients (67.9%) were women. Combined cataract surgery was performed at the time of vitrectomy in 68 eyes (81.0%), and 82 eyes (97.6%) were pseudophakic postoperatively. Preoperative EIFL staging of ERM was determined as follows: 39 eyes (46.4%) as stage 2, 33 eyes (39.3%) as stage 3, and 12 eyes (14.3%) as stage 4, respectively. Preoperatively, there were no significant differences in age and aniseikonia among the EIFL stages, but eyes with EIFL stage 4 had significantly worse BCVA and greater M-score compared with those with stages 2 or 3. For FAZ parameters, eyes with EIFL stage 2 had significantly greater FAZ area, perimeter, and circularity than those with stages 3 or 4. In 45 eyes with EIFL, namely stages 3 and 4, EIFL thickness showed significant correlation with CMT (*r* = 0.762, *P* < 0.001), BCVA (*r* = 0.332, *P* = 0.028), and M-score (*r* = 0.416, *P* = 0.006), but not with NAT (*P* = 0.050), FAZ area (*P* = 0.081), FAZ perimeter (*P* = 0.069), and FAZ circularity (*P* = 0.542).

**Table 1:** Demographics and clinical data of patients with idiopathic epiretinal membrane.

Characteristics	Total	EIFL stage						
		Stage 2	Stage 3	Stage 4	*P* ^a^	*P* (2vs3)	*P* (2vs4)	*P* (3vs4)
No. of patients (%)	84 (100%)	39 (46.4%)	33 (39.3%)	12 (14.3%)				
No. of eyes (%)	84 (100%)	39 (46.4%)	33 (39.3%)	12 (14.3%)				
Female (%)	57 (67.9%)	29 (74.4%)	19 (57.6%)	9 (75.0%)				
Age (year)	68.7 ± 7.9	68.6 ± 6.8	68.5 ± 9.1	69.3 ± 8.6	0.877			
Preoperative pseudophakia (%)	14 (16.7%)	7 (17.9%)	6 (18.2%)	1 (8.3%)				
Postoperative pseudophakia (%)	82 (97.6%)	38 (97.4%)	32 (97.0%)	12 (100%)				
BCVA (logMAR)	0.37 ± 0.20	0.32 ± 0.18	0.36 ± 0.18	0.57 ± 0.22	0.004	0.341	0.001	0.006
M-score (°)	0.40 ± 0.41	0.28 ± 0.29	0.38 ± 0.39	0.83 ± 0.53	0.001	0.334	<0.001	0.001
NAT (%)	5.25 ± 4.38	5.43 ± 4.41	4.25 ± 3.67	7.38 ± 5.66	0.356			
FAZ area (mm^2^)	0.14 ± 0.16	0.18 ± 0.12	0.13 ± 0.21	0.06 ± 0.05	<0.001	<0.001	0.001	0.765
FAZ perimeter (mm)	1.43 ± 0.94	1.69 ± 0.61	1.33 ± 1.24	0.92 ± 0.64	<0.001	0.001	0.003	0.785
FAZ circularity	0.64 ± 0.22	0.72 ± 0.08	0.57 ± 0.25	0.52 ± 0.32	0.003	0.003	0.016	0.594
CMT (μm)	431.1 ± 69.88	388.6 ± 61.17	453.2 ± 49.44	508.6 ± 48.77	<0.001	<0.001	<0.001	0.002
EIFL thickness (μm)	154.2 ± 70.16	N/A	129.9 ± 54.16	219.2 ± 68.21	N/A	N/A	N/A	<0.001

Data are presented as mean ± standard deviation.

^a^ *P*-value was calculated using Kruskal-Wallis test among stages 2, 3, and 4.

EIFL, ectopic inner foveal layer; BCVA, best-corrected visual acuity; M-score, M-chart score; NAT, New Aniseikonia Test score; FAZ, foveal avascular zone; CMT, central macular thickness.

**Fig 1:**
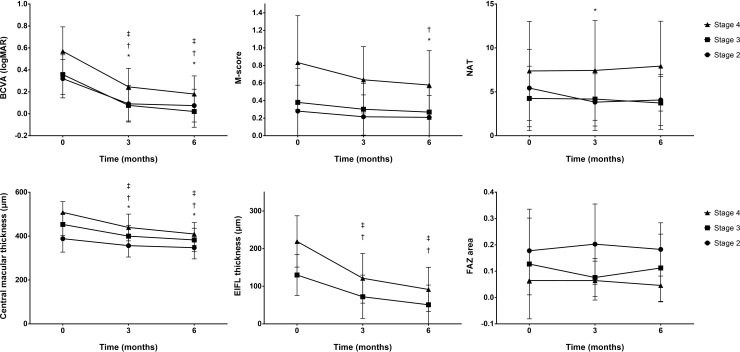
Postoperative changes in parameters according to the preoperative EIFL stage. Postoperative changes in best-corrected visual acuity (A), M-score (B), NAT score (C), central macular thickness (D), thickness of ectopic inner foveal layer (E), and area of foveal avascular zone (F). *, †, ‡ indicate significant changes from baseline in stage 2, 3, 4, respectively. BCVA, best-corrected visual acuity; M-score, M-chart score; NAT, New Aniseikonia Test score; EIFL, ectopic inner foveal layer; FAZ, foveal avascular zone.

Postoperative changes in functional and anatomical outcome parameters according to the preoperative EIFL stage are shown in Fig 1. At 6 months after surgery, the mean BCVA significantly improved in all EIFL stage groups (*P* < 0.001, *P* < 0.001, and *P* = 0.003 for stages 2, 3, and 4, respectively). The mean M-score significantly decreased in eyes with EIFL stages 2 (*P* = 0.039) and 3 (*P* = 0.011); however, it decreased in stage 4 eyes without statistical significance (*P* = 0.066). The mean NAT score did not change in eyes with EIFL stages 3 and 4, but improved in those with stage 2 EIFL at 3 months (*P* = 0.026) and 6 months (*P* = 0.053) after surgery, although statistical significance was marginal at 6 months. The mean CMT decreased significantly in all EIFL stage groups (*P* = 0.001, *P* < 0.001, and *P* = 0.005 for stages 2, 3, and 4, respectively). The mean FAZ area and FAZ perimeter showed no significant postoperative changes in any of the EIFL stage groups. The mean FAZ circularity also did not change in eyes with EIFL stages 3 and 4, but significantly improved at 6 months compared to baseline in eyes with stage 2 EIFL (0.72 ± 0.08 to 0.76 ± 0.09; *P* = 0.029). The mean EIFL thickness decreased significantly in eyes with EIFL stages of both 3 and 4 (*P* < 0.001 and *P* = 0.003, respectively).

During the 6 months after surgery, 28 of 39 eyes (71.8%) with preoperative EIFL stage 2 improved to stage 1, 10 eyes (25.6%) remained the same, and one eye (2.6%) with recurrence of ERM after removal worsened to stage 3 showing the new formation of EIFL. In 33 eyes with preoperative EIFL stage 3, 11 (33.3%) showed disappearance of EIFL at 6 months after surgery and improved to stage 1 or 2, while the others remained at the same stage. All eyes with preoperative EIFL stage 4 improved to the lower stage; however, the disappearance of EIFL was observed in only two of 12 eyes (16.7%).

Table 2 shows correlation of the postoperative EIFL stage and thickness with functional and anatomical parameters at 3 and 6 months after surgery. The EIFL stage at 3 months after surgery was significantly correlated with BCVA (*P* = 0.007), M-score (*P* = 0.003), CMT (*P* < 0.001), FAZ area (*P* < 0.001), FAZ perimeter (*P* < 0.001), and FAZ circularity (*P* = 0.016) at the same time point. The EIFL stage at 6 months after surgery had significant correlation with BCVA (*P* = 0.033), M-score (*P* = 0.008), CMT (*P* < 0.001), FAZ area (*P* < 0.001), FAZ perimeter (*P* < 0.001), and FAZ circularity (*P* < 0.001) at the same time point. However, EIFL thickness at 3 months after surgery showed significant correlation only with CMT (*r* = 0.352, *P* = 0.038) in 35 eyes with EIFL at the same time point, and EIFL thickness at 6 months after surgery showed significant correlation with BCVA (*r* = 0.370, *P* = 0.048) and CMT (*r* = 0.426, *P* = 0.021) in 30 eyes with EIFL at the same time point. In 45 eyes with EIFL preoperatively, change in EIFL thickness during 6 months after surgery was not significantly correlated with the change in the M-score (*r* = 0.218, *P* = 0.150).

**Table 2:** Correlation of functional and anatomical parameters with EIFL stage and thickness before and after the surgery.

	Baseline				At 3 months				At 6 months			
	EIFL Stage		EIFL Thickness (n = 45)		EIFL Stage		EIFL Thickness (n = 35)		EIFL Stage		EIFL Thickness (n = 30)	
	*r*	*P*	*r*	*P*	*r*	*P*	*r*	*P*	*r*	*P*	*r*	*P*
BCVA	0.309	0.004	0.332	0.028	0.294	0.007	0.170	0.330	0.238	0.033	0.370	0.048
M-score	0.338	0.002	0.416	0.006	0.323	0.003	0.304	0.075	0.297	0.008	0.218	0.257
NAT	0.018	0.890	0.367	0.050	0.177	0.187	0.373	0.105	0.072	0.596	0.202	0.421
CMT	0.623	< 0.001	0.762	< 0.001	0.659	<0.001	0.352	0.038	0.714	<0.001	0.426	0.021
FAZ area	-0.474	<0.001	-0.269	0.081	-0.708	<0.001	-0.224	0.252	-0.669	<0.001	-0.280	0.166
FAZ perimeter	-0.429	< 0.001	-0.280	0.069	-0.678	<0.001	-0.215	0.273	-0.625	<0.001	-0.287	0.156
FAZ circularity	-0.368	0.001	0.096	0.542	-0.286	0.016	0.003	0.988	-0.395	<0.001	-0.173	0.399

EIFL, ectopic inner foveal layer; BCVA, best-corrected visual acuity; M-score, M-chart score; NAT, New Aniseikonia Test score; CMT, central macular thickness; FAZ, foveal avascular zone.

Further analysis was performed to evaluate the influence of confounders on the correlation between EIFL staging and visual function. Partial correlation analysis revealed that preoperative EIFL staging remained significantly correlated with BCVA after taking into account M-score (*P* < 0.001), CMT (*P* = 0.008), outer nuclear layer (ONL) thickness (*P* < 0.001), disruption of ellipsoid zone (EZ) line (*P* < 0.001), and cotton ball sign (*P* < 0.001) and with M-score after considering BCVA (*P* < 0.001), CMT (*P* = 0.026), ONL thickness (*P* < 0.001), disruption of EZ line (*P* < 0.001), and cotton ball sign (*P* < 0.001) at the same time point. Also at 6 months after surgery, EIFL staging remained significantly correlated with BCVA after considering M-score (*P* = 0.001), CMT (*P* = 0.030), ONL thickness (*P* = 0.003), disruption of EZ line (*P* = 0.002), and cotton ball sign (*P* < 0.001) and with M-score after considering BCVA (*P* < 0.001), CMT (*P* = 0.042), ONL thickness (*P* = 0.012), disruption of EZ line (*P* < 0.001), and cotton ball sign (*P* < 0.001) at the same time point.

Preoperative EIFL stage was significantly correlated with the M-score (*r* = 0.288, *P* = 0.01), CMT (*r* = 0.422, *P* < 0.001), FAZ area (*r* = -0.492, *P* < 0.001), FAZ perimeter (*r* = -0.461, *P* < 0.001), and FAZ circularity (*r* = -0.354, *P* = 0.002) at 6 months after surgery, but not with BCVA and aniseikonia. Preoperative EIFL thickness showed a significant correlation only with CMT (*r* = 0.329, *P* = 0.036) at 6 months after surgery, but not with other parameters.

## Discussion

In this study, we investigated the changes in functional and anatomical parameters in patients who underwent PPV for the treatment of idiopathic ERM and their relationships with the EIFL staging scheme, the new OCT-based ERM severity scale described recently. Both preoperatively and during 6 months after surgery, EIFL staging showed significant association with the BCVA, metamorphopsia, CMT, and FAZ parameters, but not with aniseikonia. In eyes with EIFL, a significant correlation between EIFL thickness and metamorphopsia before surgery was not observed after surgery.

Chronic anteroposterior and centripetal traction caused by ERM may displace and reorganize the inner retinal layers, creating a continuous floor extending from the inner nuclear and inner plexiform layers across the foveal center. A novel OCT-based staging scheme of ERM based on tomographic findings related to this layer termed EIFL was proven to be effective in grading both structural changes in the macula and visual loss, as confirmed in the present study [7,10,14]. The presence of EIFL in eyes with ERM was associated with worse visual acuity, and the thickness of EIFL was negatively correlated with visual acuity [7,14]. More recent studies have shown that the EIFL grading scheme showed a good correlation not only with BCVA or CMT but also with other functional and anatomical parameters such as metamorphopsia and FAZ parameters [15,16], which was in agreement with the present study.

In addition, EIFL staging could be an easy and useful method to predict the visual prognosis after surgery. Surgical outcomes after ERM removal differ according to the EIFL stage, and surgery at earlier stages results in better visual outcomes. In a retrospective study that included 88 pseudophakic patients with idiopathic ERM, a final postoperative BCVA of ≥ 20/40 was achieved in 91.7%, 42.3%, and 5.2% of eyes with stage 2, 3, and 4, respectively [11]. Postoperative improvement in EIFL staging was more frequently observed in eyes with stage 2 compared to those with stage 3 or 4, and EIFL tended to persist in most eyes with stage 3 or 4 [7,11]. Although the thickness of EIFL decreased significantly postoperatively, visual acuity change was not associated with a postoperative decrease in EIFL thickness [7].

In patients with ERM, not only visual acuity but also functional parameters other than visual acuity including metamorphopsia and aniseikonia are important in terms of quality of vision [17–19]. A retrospective study including 60 eyes with stage 3 and 4 ERM observed a significant correlation between EIFL thickness and M-score, suggesting that EIFL might be a good indicator of metamorphopsia [16]. The results of this study also found that metamorphopsia was associated with EIFL at baseline. More importantly, the correlation between the EIFL stage and M-score was also observed at 3 and 6 months after surgery, suggesting that the EIFL stage is useful for estimating the severity of metamorphopsia both before and after removal of ERM. However, a significant correlation of EIFL thickness with metamorphopsia, which was observed preoperatively, did not persist after surgery. During postoperative follow-up, EIFL thickness was correlated only with CMT at 3 and 6 months and with BCVA at 6 months after surgery, but not with other parameters. This suggests that EIFL thickness may be less indicative of postoperative changes in macular function or anatomy compared to the EIFL stage. Once EIFL persists after ERM removal, the presence of EIFL rather than its thickness seems to have more clinical implications. Being more easily evaluable in the clinic, EIFL staging may be adopted as a simple and adequate surrogate marker for postoperative macular status after ERM removal.

To the best of our knowledge, this study is the first to investigate the association between EIFL and aniseikonia. The decrease in aniseikonia was only significant in eyes with EIFL stage 2, while those with stages 3 and 4 showed no change. Although aniseikonia caused by ERM is reported to show little or no change after surgery [20,21], the results of this study suggest that aniseikonia in eyes with early-stage ERM may improve after surgery. This is in line with a prospective study by Han et al. which found that greater improvement in aniseikonia was achieved in patients with better preoperative visual acuity [22]. In this study, EIFL had no significant relationship with aniseikonia either before or after surgery. The reason for this is elusive; however, one may hypothesize that the development of aniseikonia, particularly macropsia, in eyes with ERM may be related to the centripetal displacement of the inner retina over a large area beyond the parafoveal region [23–25], while EIFL may mainly represent the structural change at the fovea. This may result in a lower association of aniseikonia with EIFL compared to other functional parameters such as visual acuity and metamorphopsia.

The main limitation of our study was the relatively short follow-up period. However, postoperative changes in EIFL seems to occur mostly during the first 6 months after the removal of ERM, and changes during the second 6 months were reported to be minimal [7]. The strengths of our study include the prospective examination protocol in consecutive patients who underwent removal of idiopathic ERM. In addition, all surgeries were performed by a single experienced vitreoretinal surgeon, denoting a consistent surgical technique. Further studies with long-term follow-up periods are warranted to evaluate the possible associations between EIFL and functional and anatomical outcomes after ERM removal.

In conclusion, EIFL staging, the novel OCT-based grading scheme of ERM, is a good surrogate marker for visual function, including visual acuity and metamorphopsia, not only preoperatively but also during postoperative follow-up in patients with idiopathic ERM, while it was not associated with aniseikonia. In eyes with persistent EIFL after ERM removal, its thickness showed a limited association with visual function.

## Supporting information

S1 Data (XLSX)

## References

[1] Mori K, Gehlbach PL, Sano A, Deguchi T, Yoneya S. Comparison of epiretinal membranes of differing pathogenesis using optical coherence tomography. Retina. 2004;24(1):57–62. doi: 10.1097/00006982-200402000-00009 15076945

[2] Kiss CG, Barisani-Asenbauer T, Simader C, Maca S, Schmidt-Erfurth U. Central visual field impairment during and following cystoid macular oedema. Br J Ophthalmol. 2008;92(1):84–8. doi: 10.1136/bjo.2007.124016 17591669

[3] Dell’omo R, Cifariello F, Dell’omo E, De Lena A, Di Iorio R, Filippelli M, et al. Influence of retinal vessel printings on metamorphopsia and retinal architectural abnormalities in eyes with idiopathic macular epiretinal membrane. Invest Ophthalmol Vis Sci. 2013;54(12):7803–11. doi: 10.1167/iovs.13-12817 24204051

[4] Falkner-Radler CI, Glittenberg C, Hagen S, Benesch T, Binder S. Spectral-domain optical coherence tomography for monitoring epiretinal membrane surgery. Ophthalmology. 2010;117(4):798–805. doi: 10.1016/j.ophtha.2009.08.034 20045567

[5] Shiono A, Kogo J, Klose G, Takeda H, Ueno H, Tokuda N, et al. Photoreceptor outer segment length: a prognostic factor for idiopathic epiretinal membrane surgery. Ophthalmology. 2013;120(4):788–94. doi: 10.1016/j.ophtha.2012.09.044 23290984

[6] Suh MH, Seo JM, Park KH, Yu HG. Associations between macular findings by optical coherence tomography and visual outcomes after epiretinal membrane removal. Am J Ophthalmol. 2009;147(3):473–80.e3. doi: 10.1016/j.ajo.2008.09.020 19054492

[7] Govetto A, Virgili G, Rodriguez FJ, Figueroa MS, Sarraf D, Hubschman JP. FUNCTIONAL AND ANATOMICAL SIGNIFICANCE OF THE ECTOPIC INNER FOVEAL LAYERS IN EYES WITH IDIOPATHIC EPIRETINAL MEMBRANES: Surgical Results at 12 Months. Retina. 2019;39(2):347–57. doi: 10.1097/IAE.0000000000001940 29160787

[8] Joe SG, Lee KS, Lee JY, Hwang JU, Kim JG, Yoon YH. Inner retinal layer thickness is the major determinant of visual acuity in patients with idiopathic epiretinal membrane. Acta Ophthalmol. 2013;91(3):e242–3. doi: 10.1111/aos.12017 23280145

[9] Cho KH, Park SJ, Cho JH, Woo SJ, Park KH. Inner-Retinal Irregularity Index Predicts Postoperative Visual Prognosis in Idiopathic Epiretinal Membrane. Am J Ophthalmol. 2016;168:139–49. doi: 10.1016/j.ajo.2016.05.011 27210278

[10] Govetto A, Lalane RA, 3rd, Sarraf D, Figueroa MS, Hubschman JP. Insights Into Epiretinal Membranes: Presence of Ectopic Inner Foveal Layers and a New Optical Coherence Tomography Staging Scheme. Am J Ophthalmol. 2017;175:99–113. doi: 10.1016/j.ajo.2016.12.006 27993592

[11] Gonzalez-Saldivar G, Berger A, Wong D, Juncal V, Chow DR. ECTOPIC INNER FOVEAL LAYER CLASSIFICATION SCHEME PREDICTS VISUAL OUTCOMES AFTER EPIRETINAL MEMBRANE SURGERY. Retina. 2019. 10.1097/IAE.0000000000002486 30829991

[12] Ichikawa Y, Imamura Y, Ishida M. Metamorphopsia and Tangential Retinal Displacement after Epiretinal Membrane Surgery. Retina. 2017;37(4):673–9. doi: 10.1097/IAE.0000000000001232 27491043

[13] Ichikawa Y, Imamura Y, Ishida M. Associations of aniseikonia with metamorphopsia and retinal displacements after epiretinal membrane surgery. Eye (Lond). 2018;32(2):400–5. doi: 10.1038/eye.2017.201 28937146 PMC5811718

[14] Doguizi S, Sekeroglu MA, Ozkoyuncu D, Omay AE, Yilmazbas P. Clinical significance of ectopic inner foveal layers in patients with idiopathic epiretinal membranes. Eye (Lond). 2018;32:1652–60. doi: 10.1038/s41433-018-0153-9 29934636 PMC6189081

[15] Shiihara H, Terasaki H, Sonoda S, Kakiuchi N, Yamaji H, Yamaoka S, et al. Association of foveal avascular zone with the metamorphopsia in epiretinal membrane. Sci Rep. 2020;10(1):17092. doi: 10.1038/s41598-020-74190-x 33051514 PMC7555497

[16] Alkabes M, Fogagnolo P, Vujosevic S, Rossetti L, Casini G, De Cilla S. Correlation between new OCT parameters and metamorphopsia in advanced stages of epiretinal membranes. Acta Ophthalmol. 2020;98(8):780–6. doi: 10.1111/aos.14336 31902134

[17] Khanna RK, Pichard T, Pasco J, Dorvault M, Cook AR, Pisella PJ, et al. Monocular and binocular visual parameters associated to vision-related quality of life in patients with epiretinal membrane: a prospective cohort. Graefes Arch Clin Exp Ophthalmol. 2021. doi: 10.1007/s00417-020-05064-1 33394162

[18] Krarup T, Nisted I, Christensen U, Kiilgaard JF, la Cour M. Monocular and binocular end-points after epiretinal membrane surgery and their correlation to patient-reported outcomes. Acta Ophthalmol. 2020;98(7):716–25. doi: 10.1111/aos.14449 32323909

[19] Tanikawa A, Shimada Y, Horiguchi M. Comparison of visual acuity, metamorphopsia, and aniseikonia in patients with an idiopathic epiretinal membrane. Jpn J Ophthalmol. 2018;62(3):280–5. doi: 10.1007/s10384-018-0581-x 29623543

[20] Okamoto F, Sugiura Y, Okamoto Y, Hiraoka T, Oshika T. Time course of changes in aniseikonia and foveal microstructure after vitrectomy for epiretinal membrane. Ophthalmology. 2014;121(11):2255–60. doi: 10.1016/j.ophtha.2014.05.016 25012933

[21] Moon BG, Yang YS, Chung H, Sohn J. Correlation between Macular Microstructures and Aniseikonia after Idiopathic Epiretinal Membrane Removal. Retina. 2020;40(6):1160–8. doi: 10.1097/IAE.0000000000002530 30932997

[22] Han J, Han SH, Kim JH, Koh HJ. Restoration of Retinally Induced Aniseikonia in Patients with Epiretinal Membrane after Early Vitrectomy. Retina. 2016;36(2):311–20. doi: 10.1097/IAE.0000000000000731 26352554

[23] Chung H, Son G, Hwang DJ, Lee K, Park Y, Sohn J. Relationship Between Vertical and Horizontal Aniseikonia Scores and Vertical and Horizontal OCT Images in Idiopathic Epiretinal Membrane. Invest Ophthalmol Vis Sci. 2015;56(11):6542–8. doi: 10.1167/iovs.15-16874 26451682

[24] Kim JH, Kang SW, Kong MG, Ha HS. Assessment of retinal layers and visual rehabilitation after epiretinal membrane removal. Graefes Arch Clin Exp Ophthalmol. 2013;251(4):1055–64. doi: 10.1007/s00417-012-2120-7 22875136

[25] Colakoglu A, Balci Akar S. Potential role of Muller cells in the pathogenesis of macropsia associated with epiretinal membrane: a hypothesis revisited. Int J Ophthalmol. 2017;10(11):1759–67. doi: 10.18240/ijo.2017.11.19 29181322 PMC5686377

